# Titin and Troponin: Central Players in the Frank-Starling Mechanism of the Heart

**DOI:** 10.2174/157340309788166714

**Published:** 2009-05

**Authors:** Norio Fukuda, Takako Terui, Iwao Ohtsuki, Shin’ichi Ishiwata, Satoshi Kurihara

**Affiliations:** 1 Department of Cell Physiology, The Jikei University School of Medicine, Tokyo, Japan; 2 Department of Physics, Waseda University, Tokyo, Japan

**Keywords:** Calcium, cardiac muscle, connectin.

## Abstract

The basis of the Frank-Starling mechanism of the heart is the intrinsic ability of cardiac muscle to produce greater active force in response to stretch, a phenomenon known as length-dependent activation. A feedback mechanism transmitted from cross-bridge formation to troponin C to enhance Ca^2+^ binding has long been proposed to account for length-dependent activation. However, recent advances in muscle physiology research technologies have enabled the identification of other factors involved in length-dependent activation. The striated muscle sarcomere contains a third filament system composed of the giant elastic protein titin, which is responsible for most passive stiffness in the physiological sarcomere length range. Recent studies have revealed a significant coupling of active and passive forces in cardiac muscle, where titin-based passive force promotes cross-bridge recruitment, resulting in greater active force production in response to stretch. More currently, the focus has been placed on the troponin-based “on-off” switching of the thin filament state in the regulation of length-dependent activation. In this review, we discuss how myocardial length-dependent activation is coordinately regulated by sarcomere proteins.

About a century ago, Otto Frank and Earnest Starling demonstrated a fundamental principle in cardiac physiology that an increase in ventricular volume enhances the systolic performance of the heart (i.e., the Frank-Starling law of the heart; see [1] and references therein). “The Law” is a physiologically important characteristic of the heart, which regulates the cardiac output on a beat-to-beat basis. It is widely recognized that “the Law” is the same as the law of the muscle tissues [2], as both Frank and Starling understood, where active force increases in response to stretch. However, despite a number of studies having been undertaken, the molecular mechanism of length-dependent activation has not been fully understood.

First, we would like to summarize the cellular mechanisms of myocardial contraction and relaxation before discussing length-dependent activation. In cardiac muscle, contraction is regulated by micromolar concentrations of Ca^2+^ that is released from the inner membrane system [3-5]. Upon stimulation of cardiomyocytes, Ca^2+^ enters the myocyte *via *sarcolemmal L-type Ca^2+^ channels. This Ca^2+^ induces the release of Ca^2+^ from the sarcoplasmic reticulum (SR) [i.e., Ca^2+^-induced Ca^2+^ release mechanism], resulting in the binding of Ca^2+^ to troponin C (TnC) and the subsequent formation of cross-bridges [i.e., actomyosin (actin-myosin) interaction]. Unlike in skeletal muscle, cardiac myofilaments are not normally fully activated because the intracellular Ca^2+^ concentration ([Ca^2+^]_i_) is maintained relatively low (~10^-6^ M) in systole [3]. For relaxation to occur, four transport systems are involved to lower [Ca^2+^]_i_: (1) sequestration into the SR by the Ca^2+^-ATPase pump (i.e., SERCA2a protein), (2) efflux *via *the sarcolemmal Na^+^/Ca^2+^ exchanger, (3) extrusion by the sarcolemmal Ca^2+^-ATPase pump, and (4) uptake into mitochondria *via *the Ca^2+^ uniporter. Myofilament relaxation takes place following dissociation of Ca^2+^ from TnC.

When stretched under physiologic conditions, mammalian cardiac muscle exhibits a biphasic force response to stretch, in that twitch force increases immediately after stretch, followed by a slow and gradual increase in the force. In a pioneering study, Allen and Kurihara [6] used the Ca^2+^-sensitive photoprotein aequorin and simultaneously measured [Ca^2+^]_i_ and twitch force in living cardiac muscle. It was demonstrated that the rapid first phase occurs independently of [Ca^2+^]_i_, and the second phase is coupled with a rise of [Ca^2+^]_i_ (i.e., “slow effect”). It is now recognized that the former is controlled by myofilaments and it is responsible for the beat-to-beat basis modulation of the mechanical response to stretch, predominantly underlying the Frank-Starling mechanism. For the slow effect, the following mechanisms have been proposed. First, Ruknudin *et al. *[7] reported that stretch-activated channels capable of generating inward currents (including Ca^2+^) may operate to increase [Ca^2+^]_i_. Second, Alvarez *et al. *[8] provided evidence for stretch-induced activation of the sarcolemmal Na^+^/H^+^ exchanger by local autocrine/paracrine systems involving angiotensin II and endothelin-1, resulting in an increase in [Na^+^]_i_ and a subsequent rise of [Ca^2+^]_i_ (reviewed in [9]). Third, Petroff *et al. *[10] demonstrated that stretch induces PtdIns-3-OH kinase-dependent phosphorylation of both Akt and the endothelial isoform of nitric oxide synthase, nitric oxide production and an increase in Ca^2+^-spark frequency. Therefore, the stretch-induced production of nitric oxide may enhance the release of Ca^2+^ from the SR. In this article, we will focus on the rapid component of the force response after stretch, and discuss how this is integrated in the supramolecular structure sarcomere.

Another important finding by Allen and Kurihara [6] is that a quick release of muscle length from *L*_max_ (where twitch force reaches the maximum) to ~90% *L*_max_ at various points of isometric twitch contraction causes a transient increase in [Ca^2+^]_i_ (i.e., extra-Ca^2+^). Subsequent studies revealed that extra-Ca^2+^ is in proportion to the magnitude of force reduction [11], and is indeed markedly diminished in the presence of the actomyosin inhibitor 2, 3-butanedione monoxime (BDM) [12]. It is therefore considered that cross-bridge formation (or dissociation) enhances (or reduces) Ca^2+^-binding to TnC *via *a feedback mechanism, and both complementarily affect each other during twitch contractions. However, recent studies suggest that this is not the sole mechanism involved in length-dependent activation.

The first direct evidence that myofibrillar contractility is increased with length in cardiac muscle was provided by Hibberd and Jewell [13] in experiments with chemically skinned preparations. They observed that active force increases with sarcomere length at submaximal levels, as well as at maximal levels, resulting in the leftward shift of the force-pCa (= –log [Ca^2+^]) curve (i.e., increase in “Ca^2+^ sensitivity of force”). This property has been confirmed by a number of researchers, and is now widely recognized to underlie length-dependent activation in cardiac muscle under physiologic conditions.

A straightforward interpretation that can seemingly account for the findings by Hibberd and Jewell [13] may be that TnC plays a role as a “length sensor” that changes its affinity for Ca^2+^, depending on the length of the sarcomere. This proposal is based on the observation that the length-dependent shift of the force-pCa curve in skinned cardiac muscle is diminished after exchange of TnC for the isoform taken from fast skeletal muscle that shows less length dependency [14, 15]. Later, however, the group of Moss challenged this proposal providing strong evidence that (1) length-dependent activation in skeletal muscle is unaltered after TnC is exchanged for the cardiac isoform [16], and (2) overexpression of skeletal TnC in cardiomyocytes does not alter the length-dependent activation [17]. Also, the findings that lengthening increases (1) maximal Ca^2+^-activated force (e.g., [13, 18-20]) and (2) Ca^2+^-independent, rigor cross-bridge-dependent active force [21, 22] render this proposal further unlikely.

Instead, it has been proposed that the leftward shift of the force-pCa curve results from an increase in the fraction of cross-bridges coupled with structural changes in the sarcomere. It is now widely accepted that the actomyosin interaction is a stochastic process (e.g., [23]). It is therefore considered that the attachment of myosin heads to the thin filament is enhanced as the distance between the thick and thin filament is reduced, as simulated by Ishiwata and colleagues based on a mathematical model [24, 25]. Indeed, osmotic compression reduces the lattice spacing and increases contractile force [18, 26, 27], coupled with enhanced cross-bridge formation [28]. Also, studies with synchrotron X-ray have provided direct evidence that the lattice spacing is reduced in response to stretch within the physiological sarcomere length range (i.e., ~1.8 – ~2.4 μm) in various animal species (e.g., [29, 30]). These findings are consistent with the notion that the lattice spacing plays a pivotal role in length-dependent activation (e.g., [18, 26, 27]).

What triggers lattice spacing reduction in cardiac muscle at the stretched state? Does it occur intrinsically in the sarcomere or with a support from the extracellular component such as the collagen network (as suggested in [19])? Recent studies have revealed that the giant sarcomere protein titin (also known as connectin) is involved in the modulation of the lattice spacing (e.g., [31, 32]). Titin is a muscle-specific elastic protein (3–4 MDa) that spans from the Z-line to the M-line in half-sarcomere ([33]; see also recent reviews [34-39]). Titin develops passive force when stretched by external force, resulting from extension of its spring element in the I-band region of the sarcomere (for recent reviews and original citations; [34-39]). While titin’s A-band portion is composed of relatively simple patterns of immunoglobulin (Ig)-like and fibronectin type 3 (Fn3) repeats, the I-band region has a complex sequence with distinct extensible segments: the tandem Ig segments (tandemly arranged Ig-like domains), the PEVK segment [rich in proline (P), glutamate (E), valine (V) and lysine (K)] and the segment that has a unique amino acid sequence (N2A or N2B). In cardiac muscle, two types of titin exist, i.e., stiff N2B titin (containing the N2B segment) and less stiff N2BA titin (containing both N2B and N2A segments) (Fig. **1**). N2BA titin contains an additional middle Ig segment, the N2A segment, and the PEVK segment of variable lengths. Therefore, its passive force is lower than that of N2B titin. The expression level of N2B and N2BA titins varies in a species-specific and location-specific manner, but both isoforms are expressed at similar levels in the ventricle of most mammals, including humans [40, 41].

Two articles published in 2001 have significantly expanded our understanding of length-dependent activation by providing evidence that titin is involved in length-dependent activation. First, Cazorla *et al. *[42] measured force-pCa curves in skinned rat cardiomyocytes at different levels of passive force, modulated by a pre-history of stretch, and found that the magnitude of the sarcomere length-dependent increases in Ca^2+^ sensitivity of force varies in response to passive force, rather than to sarcomere length. They also found that interfilament lattice spacing is expanded in response to a decrease in titin-based passive force. Titin binds to actin in and near the Z-line [43, 44]. Therefore, titin runs obliquely (not in parallel to the thin filaments) in the sarcomere, resulting in the production of radial force as well as longitudinal force in the lattice; the former force may pull the thin filament closer to the thick filament (Fig. **2**). This likely increases the likelihood of myosin binding to actin. In the myofilament lattice, the electrostatic force (F_es_) arising from the negative charge carried by thick and thin filaments tends to separate these filaments. Cazorla *et al. *[42] calculated the values of F_es_ at various sarcomere lengths and concluded that titin-based radial force and F_es_ are of similar magnitude in the cardiac myofilament lattice, at least in relaxing conditions. It is therefore likely that titin develops radial force that is large enough for it to play a role in counteracting F_es_. Secondly, Fukuda *et al. *[19] applied limited trypsin treatment to rat ventricular trabeculae to selectively degrade titin, and found that the slope of the length-force curve became less steep. Since attenuation of the length-dependence was observed under conditions where the lattice spacing (as indexed by muscle width) was not significantly changed, it was concluded that a mechanism operating independent of the lattice spacing modulation was responsible. It has been reported that titin is an integral component of the thick filament, tightly binding to myosin and myosin-binding protein C [45]. Therefore, sarcomere extension by external force may cause mechanical strain of the thick filament *via *titin’s longitudinal force, allowing myosin to attach to actin. Indeed, Wakabayashi *et al. *[46] reported that in frog skeletal muscle, sarcomere extension causes an increase in the myosin periodicity, accompanied by a loss of characteristic resting order of myosin heads around the thick filament backbone. Thus, a similar mechanism may operate in cardiac muscle within the physiological sarcomere length, based on the higher level of stiffness compared to skeletal muscle. It is therefore reasonable to consider that titin modulates length-dependent activation and that the mechanism includes titin-based modulation of the lattice spacing and the thick filament structure [30-32, 47].

β-Adrenergic stimulation induces activation of protein kinase A (PKA), resulting in the phosphorylation of various proteins in cardiomyocytes, causing marked alteration in systolic and diastolic performances of the heart (see [3]). It has been reported that PKA phosphorylates the N2B sequence of titin (both N2B and N2BA isoforms), resulting in a decrease in passive force, especially in the physiological sarcomere length range [48, 49]. Therefore, it may be possible that length-dependent activation is attenuated by PKA, as pointed out elsewhere [22]. 

However, the group of de Tombe challenged the lattice spacing hypothesis, providing evidence that the lattice spacing and Ca^2+^ sensitivity of force are not well correlated [50]. Therefore, factors other than the lattice spacing are likely involved in the regulation of length-dependent activation, obtained at the steady-state. Indeed, length-dependent activation reportedly varies as a function of *recruitable* (i.e., resting) cross-bridges that have ATP and can, therefore, potentially produce force upon attachment to the thin filament [31, 32]. This notion is supported by the experimental evidence from different groups showing that the length dependency is attenuated at high cooperative activation states with a non-force-generating analogue of myosin subfragment 1 (NEM-S1) [51] or MgADP [18] and it is enhanced at low cooperative activation states with inorganic phosphate or H^+^ [20]. Therefore, the “on-off” equilibrium of the thin filament state, which is regulated by Tn under physiologic conditions, likely determines the magnitude of length-dependent activation in concert with titin-based regulation. 

Tn is a heterotrimer of distinct gene products: i.e., TnC (a Ca^2+^ receptor), TnI (an inhibitor of actomyosin interaction that tightly binds to either actin or Ca^2+^-TnC) and TnT (an anchor that binds to tropomyosin, TnI and TnC) (e.g., [52-55]). Two metal binding sites exist in the C-terminal domain of TnC that bind both Mg^2+^ and Ca^2+^ with relatively high affinity. However, due to the higher concentration of Mg^2+^ under physiologic conditions (~1 mM compared with ~0.1 – ~1 μM for Ca^2+^), these sites are normally occupied by Mg^2+^, during both diastole and systole. Cardiac (fast skeletal) TnC has one (two) regulatory Ca^2+^-binding site(s) with relatively low affinity in the N-terminal domain of this molecule. When [Ca^2+^]_i_ is increased during systole, Ca^2+^ binds to the regulatory Ca^2+^-binding site, resulting in the onset of the conformational change of the thin filament. During diastole, the C-terminal domain of TnI tightly binds to actin, and tropomyosin blocks the actomyosin interaction (“off” state). However, when Ca^2+^ binds to the regulatory Ca^2+^-binding site of TnC during systole, the C-terminal domain of TnI is dissociated from actin and binds to the N-terminal domain of TnC, due to the enhanced TnC-TnI interaction (“on” state). It is generally considered that the transition from the “off” to “on” state is associated with a movement and rotation of tropomyosin on the thin filament [56, 57]. Previous studies have demonstrated that the “on-off” equilibrium depends on the isoform of Tn subunits (e.g., [54]), as well as on the fraction on the strongly bound cross-bridges, such as the actomyosin-ADP complex [58]. 

A mouse model expressing slow skeletal TnI (that lacks cardiac-specific PKA phosphorylation sites; ssTnI) in the ventricle has been widely used to investigate the physiological properties of TnI under various conditions (e.g., [59]). Ventricular muscle expressing ssTnI shows greater Ca^2+^ sensitivity of force than control muscle, due presumably to the acceleration of the transition from the “off” state to the “on” state of the thin filament [59, 60]. By using this mouse model, the group of Solaro [60] demonstrated that there was less length-dependent activation in ventricular muscle expressing ssTnI than in control muscle, associated with an increase in Ca^2+^ sensitivity of force. Later, by taking advantage of a quasi-complete Tn exchange technique, Terui *et al. *[61] provided strong evidence that the “on-off” equilibrium of the thin filament state plays an important role in length-dependent activation. The authors demonstrated that replacement of endogenous Tn with exogenously added rabbit fast skeletal Tn (sTn) attenuated the length dependency in skinned porcine ventricular muscle, to a magnitude similar to that observed in rabbit fast skeletal muscle (see also [62] for differing magnitudes of length-dependent activation in cardiac and skeletal muscles). Considering the finding that Ca^2+^ sensitivity of force is increased in association with enhanced cross-bridge formation upon replacement with rabbit fast skeletal Tn [61], the switching of Tn from the cardiac to fast skeletal isoform likely accelerates the transition from the “off” state to the “on” state of the thin filament (similar to the finding in [60]). It is therefore considered that length-dependent activation is attenuated as a result of a decrease in the fraction of *recruitable* cross-bridges. Also, Terui *et al. *[61] demonstrated that an increase in titin-based passive force, induced by manipulating the pre-history of stretch (as in [30]), enhanced length-dependent activation, in both control and sTn-reconstituted cardiac muscles, suggesting that this phenomenon is coupled with both titin-based passive force and “on-off” switching of the thin filament state, with titin-based passive force acting as a trigger in this phenomenon.

In conclusion, myocardial length-dependent activation is the basis for the Frank-Starling mechanism of the heart, which is modulated by sarcomere proteins, rather than *via *a rise in [Ca^2+^]_i_. A simultaneous measurement of [Ca^2+^]_i_ and twitch force in intact cardiac muscle revealed a tight coupling between Ca^2+^-binding to TnC and cross-bridge formation, both complimentarily affecting each other. Recent studies with skinned myocardial preparations demonstrated that at the steady-state, length-dependent activation is primarily regulated *via *“on-off” switching of the thin filament state in concert with titin-based lattice spacing modulation. Therefore, under physiologic conditions, cross-bridges recruited *via *this mechanism may enhance Ca^2+^-binding to TnC and consequently recruit more cross-bridges. Future work is warranted to fully elucidate the enigmatic phenomenon of length-dependent activation.

## Figures and Tables

**Fig. (1):**
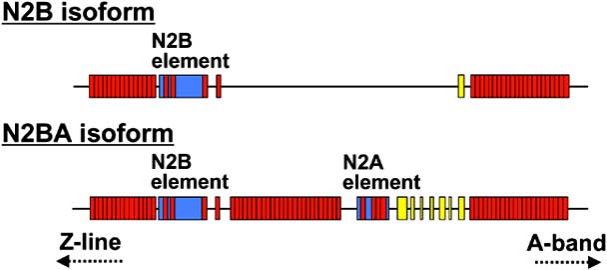
Splice isoform diversity in the extensible regions of N2B and N2BA titin isoforms. Both isoforms have constitutive Ig domains and PEVK residues near the A-band. Red, Ig domain; yellow, PEVK domain; blue, unique sequence. Based on [39].

**Fig. (2):**
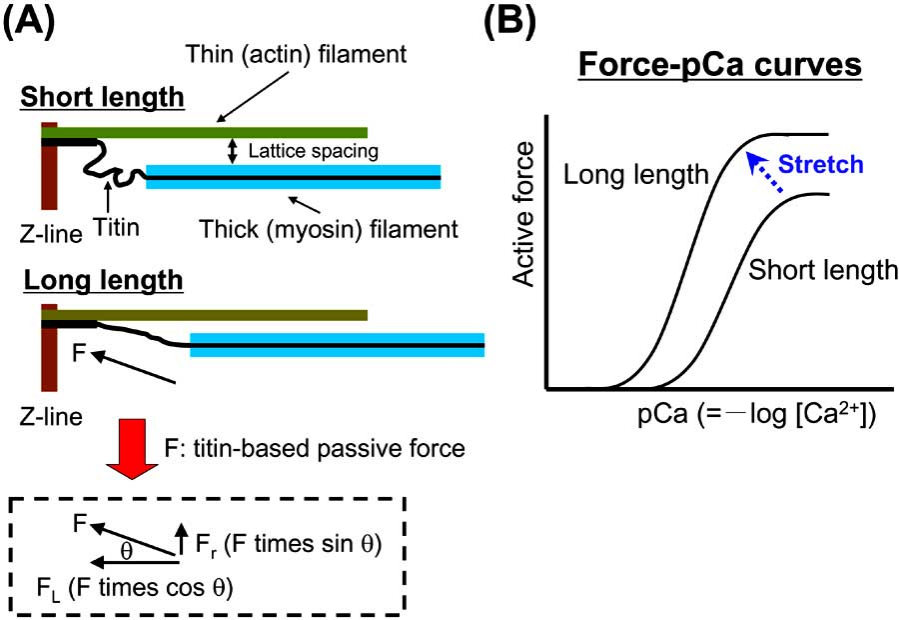
Schematic illustration showing the role of titin in the modulation of interfilament lattice spacing (**A**) and length-dependent changes in the force-pCa curve (**B**). At a long length, titin produces passive force (F) in the myofilament lattice. The force is divided into longitudinal (F_L_) and radial (F_R_) components, the latter of which reduces interfilament lattice spacing. The lattice reduction is likely to enhance myosin attachment to actin and, consequently, it causes a leftward shift of the force-pCa curve and increases the maximal Ca^2+^-activated force [right, see arrow in (**B**)].
